# The nasal microbiota of dairy farmers is more complex than oral microbiota, reflects occupational exposure, and provides competition for staphylococci

**DOI:** 10.1371/journal.pone.0183898

**Published:** 2017-08-29

**Authors:** Sanjay K. Shukla, Zhan Ye, Scott Sandberg, Iris Reyes, Thomas R. Fritsche, Matthew Keifer

**Affiliations:** 1 Molecular Microbiology Laboratory, Center for Human Genetics, Marshfield Clinic Research Institute, Marshfield, Wisconsin, United States of America; 2 Biomedical Informatics Research Center, Marshfield Clinic Research Institute, Marshfield, Wisconsin, United States of America; 3 National Farm Medicine Center, Marshfield Clinic Research Institute, Marshfield, Wisconsin, United States of America; 4 Division of Laboratory Medicine, Marshfield Clinic, Marshfield, Wisconsin, United States of America; 5 VA Puget Sound, Seattle, Washington, United States of America; Universitatsklinikum Munster, GERMANY

## Abstract

Allergic and autoimmune diseases had been attributed to lack of exposure to biodiversity, an important factor in regulating immune homeostasis in a healthy host. We posit that the microbiome of healthy dairy farmers (DF) will be richer than non-farmers (NF) living in urban settings due to exposure to a greater biodiversity in the dairy environment. However, no studies have investigated the relationships between microbiota of dairy farmers (DF) compared with urban non-farmers (NF). We compared the nasal and oral microbiota of dairy farmers (N_DF, O_DF, respectively) with nasal and oral microbiota of NF in the same geographical area. The N_DF showed high microbial diversity with hundreds of unique genera that reflected environmental/occupational exposures. The nasal and oral microbiomes clustered separately from each other using Principal Coordinate Analysis, and with DF harboring two-fold and 1.5-fold greater exclusive genera in their nose and mouth respectively, than did non-farmers. Additionally, the N_DF group had a lower burden of *Staphylococcus* spp. suggesting a correlation between higher microbial diversity and competition for colonization by staphylococci. The N_DF samples were negative for the *mecA* gene, a marker of methicillin-resistance in staphylococci. The lower burden of staphylococci was found to be independent of the abundance of *Corynebacterium* spp. Exposure to greater biodiversity could enhance microbial competition, thereby reducing colonization with opportunistic pathogens. Future studies will analyze whether exposure to livestock microbiomes offers protection from acute and chronic diseases.

## Introduction

Healthy humans are known to carry a diverse microbial consortium, whereas decreased microbial diversity is observed in certain disease states including obesity, inflammatory bowel disease, and diarrhea associated with gastrointestinal infections [1–4]. Furthermore, increases in prevalence of allergic and autoimmune diseases have also been attributed to the lack of exposure to diverse microbial antigens, an important factor in regulating immune homeostasis in a healthy host [5]. Decreased microbial diversity can occur in response to antibiotic use [6], certain diets, or when living in an environment that lacks biodiversity [7] with limited exposure to non-pathogenic organisms. Exposure to non-pathogenic microbes, particularly during childhood, is felt to be important for developing and maintaining a rich microbiota (i.e., the hygiene hypothesis) [8].

Furthermore, the composition of human microbiota is shaped by a variety of intrinsic and extrinsic factors. Intrinsic factors include, but are not limited to, the overall health of an individual, an individual’s susceptibility to chronic disease (i.e., genetic factors), disease status, presence of a chronic disease, and progression to a chronic disease [9–15]. Extrinsic factors include exposure to antibiotics, diet, exercise, and fatigue besides environmental conditions specific to an individual’s environment [16–20]. These factors not only provide direct exposure of an individual to a community of microorganisms, but may also modulate the composition of a microbiome through the production of cytokines and other biologically active molecules that exert selective pressures. Understanding the human microbiome in people of different races, diseases, and occupations, and their interaction with host genetics, could potentially advance the field of personalized and precision medicine [21–24].

Different occupations have differing work environments that are expected to have some effect on host physiology, immune system, and microbiome. Since dairy farmers spend a considerable amount of time in barns and fields with long work hours, it is expected that they would be exposed to environments having a different biodiversity than non-farming environments. Dairy industry workers are engaged in activities including feeding and milking of cows and cleaning up manure on a daily basis. These workers are exposed to microorganisms and airborne dust known to cause respiratory diseases, including large (>3μ) and small (<3μ) particles such as fungal spores and bacteria, respectively [25,26]. The daily working environment of dairy farmers is considerably different from that of workers in other agricultural and non-agricultural industries. Little is known, however, about the microbiomes associated with dairy farmers and their effect on nasal colonization by staphylococci in general and *Staphylococcus aureus*, an opportunistic pathogen in particular. *S*. *aureus* and *S*. *epidermidis* are reported to colonize the human nares at the rate of 20 to 30% and >90% of individuals respectively [27,28]. Nasal colonization of *S*. *aureus* increases the risk for diseases caused by the same bacterium. A study of bacterial assemblages along the nasal passages by Wos-Oxley et al reported the presence core bacterial community regardless of whether they have chronic nasal inflammation (CNS) or not [29]. Further, a study by Camarinha-Silva et al showed that up to 85% of nasal bacterial community was shared among two unrelated population [30]. However, Yan et al reported spatial variation in nasal microbial community along with *S*. *aureus* carriage modulated by species of *Corynebacterium* [31].

It has been reported that children growing up in agricultural environments are less susceptible to asthma and allergies and more protected from allergic sensitization than those growing up in urban areas [32]. However, the number of studies that have investigated associations between environmental microbiomes and disease susceptibility in rural and urban populations are limited [5,32,33].

The goal of this study was to identify and characterize the baseline nasal and oral microbiota of dairy farmers and compare these with the nasal and oral microbiota of non-farmers. We predicted that the nasal and oral microbiota of dairy farmers would be more diverse than the microbiota of non-dairy control subjects, reflecting occupational and environmental exposure.

## Materials and methods

### Recruitment

This study was approved by the Marshfield Clinic Research Foundation Institutional Review Board. Since Wisconsin is a dairy state with over 9,900 working farms as of 2015, we decided to collect samples from dairy farmers only rather than combining samples from crop and livestock farmers (http://www.wmmb.com/assets/images/pdf/WisconsinDairyData.pdf). We recruited 21 dairy farmers and 18 non-farm workers older than 18 years-of-age from central Wisconsin for this study. A dairy farmer was defined as a person who currently worked on a dairy farm for at least 40 hours per week. A non-farmer was someone working full-time in an office-based job and not living on any kind of farm. A non-farmer had not worked in a dairy farm or participated in other types of agriculture in the last ≥5 years. All non-farmers had office-based occupations at the time of study. All samples were collected in Wisconsin, USA in June 2014. In particular, farm workers were recruited from two dairy farms in an area with a two-mile radius in Marathon County, Wisconsin and their samples were collected onsite in the middle of a work day. The dairy farms primarily milked Holstein cows, the most common cattle breed in the US. Nine participants were recruited from one farm and 12 were recruited in another. There were 18 non-farm workers who were recruited from one non-farm organization and their samples were collected in the middle of a work day from a neighboring County. The two groups were roughly 11 miles apart. All subjects consented to study participation.

Microbiota samples were collected using the ESwab kit from Copan Diagnostics, Inc. (Murrieta, CA) during the months of March and April in 2014. Two swabs were used to collect samples. One swab was used to collect materials from saliva and buccal surfaces, and the other swab was used to collect surface materials from an anterior nasal passage. Two complete clockwise circular motions with the swab in the nose and mouth were implemented to collect the clinical material. The samples were transferred in a container with dry ice immediately, brought to the lab within an hour and then stored at -80°C until further processing. The samples were coded in the following manner: oral samples from dairy farmers (O_DF), oral samples from non-farmers (O_NF), nasal samples from dairy farmers (N_DF), and nasal samples from non-farmers (N_NF). Any individual taking antibiotics or with a history of taking antibiotics in the last 3 months was excluded from the study. All subjects provided informed verbal consent as approved by the Marshfield Clinic Institutional Review Board.

### Molecular methods

The nasal and oral samples stored in 200 μl of the transport buffer (ESwab kit, Murrieta, CA) were thawed and vortexed for 20 seconds. The DNA was extracted from the swabs using the QiAmp DNA blood mini kit (Qiagen Inc; Germanton, MD) and quantified using the Qubit dsDNA HS assay kit (Life Technologies, Grand Island, NY). All barcodes were synthesized by Integrated DNA Technologies (Ames, IA). The V4 region of the 16S rRNA bacterial gene was amplified with barcoded (along with Illumina adapter) primers using 515F and 806R primer pairs by following the protocol as described in Caporaso et al. [34]. The PCR was done in a PE9700 Thermocycler for 30 cycles under the following conditions: 94°C for 2 min, 30 cycles of 94°C for 45 seconds, 61°C for 45 seconds, and 72°C for 45 seconds, followed by a 10 min hold at 72°C. The concentration of amplicons was normalized using the SequalPrep normalization plate (Life Technologies, NY). The amplicons were sequenced using the MiSeq Reagent Kit V2 Sequencing Primers described in Caporaso et al. [34].

*Microbiome analysis*. Sequence analysis was performed utilizing the modules of the Mothur software (version 1.34, www.mothur.org) [35]. Sequences were screened for ambiguity (maximum ambiguity allowed: 0) and homopolymers (maximum homopolymer length allowed: 4 nucleotides). Chimeras were detected using the Uchime algorithm developed by Edgar et al. [36], and singleton sequences were removed from the original fasta file for creating a manageable yet meaningful OTU table for downstream analyses, particularly for calculating pairwise distances between aligned sequences [35]. The sequences were clustered as OTUs using the threshold of 3% divergence (97% pairwise identity cutoff). All taxonomic classifications were assigned using the naïve Bayesian algorithm developed for the RDP classifier, as in Mothur 1.3.4 [35]; OTU representative sequences were classified at the taxonomic level by comparing them with sequences from the SILVA database with the threshold limit of sequence homology set to 80%. Sequence abundance for each sample was calculated to build an OTU table. OTU data were analyzed at the phylum, family, and genus levels by implementing >1% relative abundance cut off across the three taxonomic levels. However, in situations where a phylum, a family, or a genus were represented by <1% relative abundance in a different sample group (e.g. N_NF or N_DF, etc.) but one group was >1%, both data were provided in tables. These comparisons were done to understand microbial landscape of dairy farmers and non-farmers at the major and minor taxonomic levels.

The OTU table was used to calculate descriptive indices for microbial diversity (alpha-diversity, non-parametric Shannon index), richness (Chao1 richness estimate), and phylogenetic evenness of the microbiome species (Shannon index-based measure of evenness). Variation in the alpha-diversity among samples was tested with *T*-test for individual microbiomes of subjects due to inter-individual differences existing between microbiomes. Paired comparisons were performed with post-hoc Student’s t-test to identify significant differences among the sample groups [13]. Beta-diversity measures were performed using AMOVA to compare the species composition among the sampled microbiota from dairy farmers and non-dairy farmers. Principal coordinate analysis (PCoA) was performed directly on the OTU table to analyze within-group relationships between oral and nasal microbial species. Distance matrices were constructed based on the index of similarity of community composition with distances and structure estimated with the Bray-Curtis indices, respectively. These changes were visualized by PCoA plot with the first PCo on the x-axis and second PCo on the y-axis. Descriptive statistics of sequences and relative abundance were calculated and visualized in boxplots using ggplot2 package in R [http://www.R-project.org/]. A *T*-test was performed to determine the significant differences of relative abundance at the phylum, family, and genus level of data from the four groups. All statistical analyses were performed with MOTHUR 1.34.0 and R 3.1.0 [35] (http://www.R-project.org/). We have reported both *p*-values as well as adjusted *p*-values adjusted for false discovery rates.

*mecA PCR*. Extracted DNA from each sample was tested by three *S*. *aureus* specific *mecA* (codes for methicillin-resistance) primers. Briefly, three primer pairs, *mecA*F1-*mecA*R1, *mecA*F2-*mecA*R2, and *mecA*F3-*mecA*R3 that covered the entire *mecA* gene were used to determine the presence of *mecA* gene. The 25 μl master mix included 12.5 μl of HotStar *Taq* polymerase, 20 pmol of each of forward and reverse primers, 8.5 μl of water, 2 μl of the templated DNA was subjected to 30 cycles of denaturation at 94°C for 30 sec, 48°C for 30 sec, and 72°C for 60 sec (see S1 Table) [37].

## Results

### Phenotypic characterization of subjects

All subjects were recruited from Wisconsin. There were 21 dairy farmers and 18 non-farmers included in the study. Subject demographics and their dairy activities are described in Table 1. The age range of both dairy farmers and non-farmers was 20 to 64 years. Sixty percent of the dairy farmers worked more than 40 hours per week on the farm. All or nearly all dairy farmers had current daily occupational exposure to livestock, manure, and hay in their workplace, while none of the non-farmers had that exposure. None of the non-farmers had worked on a farm for the last 5–10 years. Non-farmers had household pets such as cats and dogs, but they did not have cattle, horses, or rodents. Only one non-farming individual (5.3%) reported taking less than one shower per day, while seven farmers (33.3%) reported taking less than one shower per day. Thus, occupational as well as personal microbial exposures differed between farmers and non-farmers in our sample population.

**Table 1:** Subject demographics and their environmental exposures.

Subject	Dairy farmer *n* (%)	Non-farmer *n* (%)	*p*-value
**Age Range**			.41
20–24	3 (14)	2 (11)	
25–34	4 (19)	6 (33)	
35–44	6 (29)	4 (22)	
45–54	3 (14)	5 (28)	
55–64	5 (24)	1 (6)	
**Occupational exposure**			
Livestock	20 (95)	0 (0)	< .0001
Manure	21 (100)	0 (0)	< .0001
Hay	15 (79)	0 (0)	< .0001
Tractor operation	15 (75)	0 (0)	< .0001
**Work hours/week**			.007
≤ 40	8 (40)	15 (83)	
> 40	12 (60)	3 (17)	
**Showers/day**			.03
< 1	7 (33)	1 (6)	
≥ 1	14 (67)	17 (94)	
**Household pets**			
Mammals			
Cat	3 (14)	7 (39)	.07
Dog	3 (14)	7 (39)	.07
Cattle	1 (5)	0 (0)	.54
Horse	2 (10)	0 (0)	.28
Rodent (gerbil)	1 (5)	0 (0)	.54
Rabbit	1 (5)	0 (0)	.54
Fish	1 (5)	0 (0)	.54
Bird (chicken)	1 (5)	0 (0)	.54
Reptile			
Turtle	1 (5)	0 (0)	.54
Lizard	0 (0)	1 (6)	.46

### Microbiota analyses at the phylum, family, and genus level

We sampled the oral and nasal mucosa to determine the respective microbiota from the four targeted groups: nasal dairy farmer (N_DF), oral dairy farmer (O_DF), nasal non-farmer (N_NF), and oral non-farmer (O_NF) using the V4 region of the 16S rRNA gene. A total of 9,846,074 sequences were generated from 78 clinical samples, of which 9,251,196 sequences met the quality control standard. An average of 118,605 reads was analyzed for each sample. However, the average number of reads from the four groups ranged from 110,386 in N_DF to 135,822 N_NF. The Good’s coverage was >0.992 for all samples. Operational taxonomic units (OTUs) were assigned to phyla, family, and genus taxonomic levels, and comparative analyses were also performed for these three taxonomic categories.

### Nasal microbiome of dairy farmers was richer and diverse

**Fig 1:**
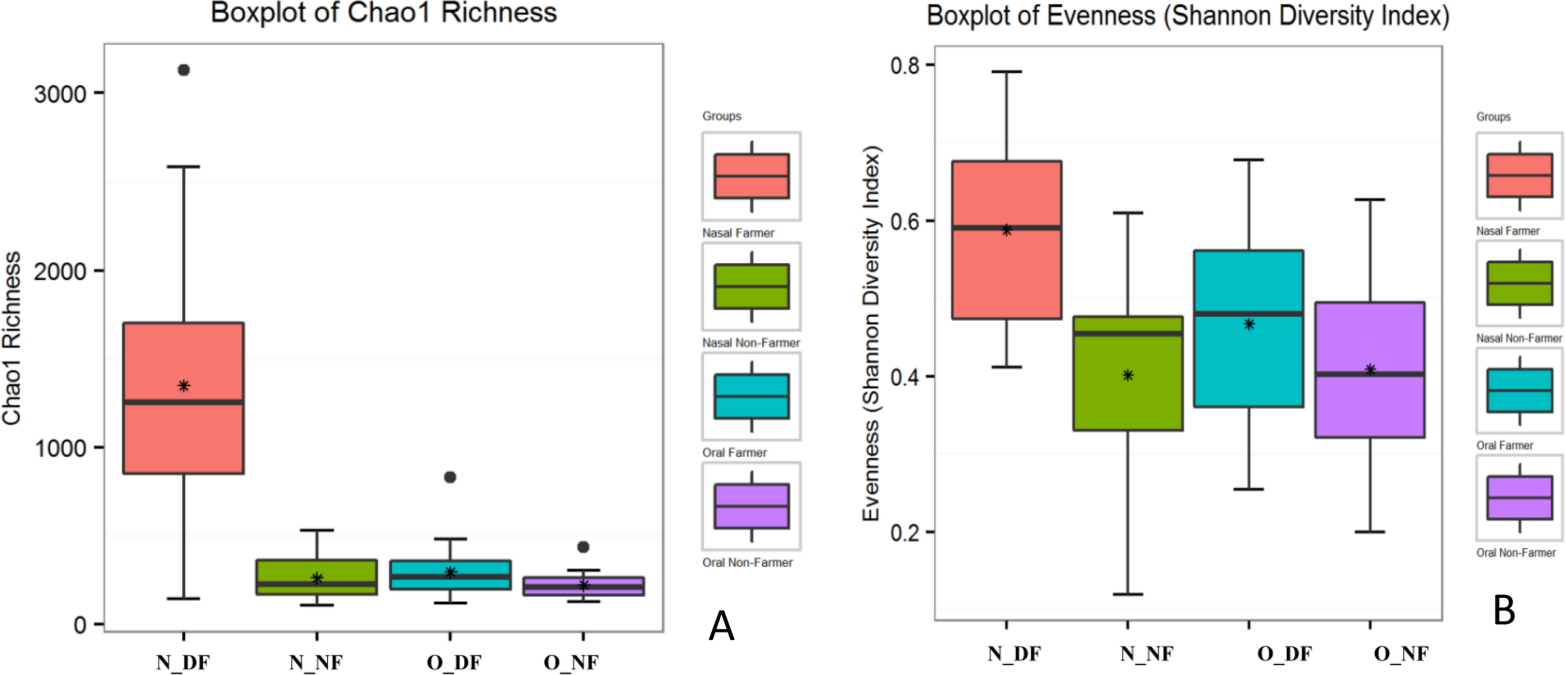
**A.** Boxplot representation of Chao 1 richness in N_DF, N_NF, O_DF, and O_NF groups (N_DF = Nasal dairy farmer; N_NF = Nasal non-farmer; O_DF = Oral dairy farmer; O_NF = Oral non-farmer). **Fig 1B.** Boxplot representation of Shannon diversity index (evenness) for N_DF, N_NF, O_DF, and O_NF groups (N_DF = Nasal dairy farmer; N_NF = Nasal non-farmer; O_DF = Oral dairy farmer; O_NF = Oral non-farmer).

Among the four sample groups, nasal microbiome from dairy farmers (N_DF) showed maximum species richness compared with that of the other three sample groups: N_NF, O_DF, O_NF. Specifically, the Chao 1 richness was significantly higher in N_DF when compared to N_NF (*p*-value = 1.55E-07), to O_NF (*p*-value = 1.24E-07) and to O_DF (*p*-value = 1.03E-05) individually (Fig 1A). However, the Chao 1 richness in the three groups—N_NF, O_DF, and O_NF—were not significantly different from each other. Similarly, differences in Shannon Diversity index was significant between N_DF and N_NF (*p*-value = 2.90E-05), between N_DF and O_DF (*p*-value = 0.002), and between N_DF and O_NF (*p*-value = 3.51E-05) (Fig 1B). However, species diversity was not significantly different between N_NF and O_NF (*p*-value = 0.092) nor for N_NF vs O_DF (*p*-value = 0.102). Oral samples did not have as much microbial diversity as nasal samples regardless of farming or non-farming status.

**Fig 2:**
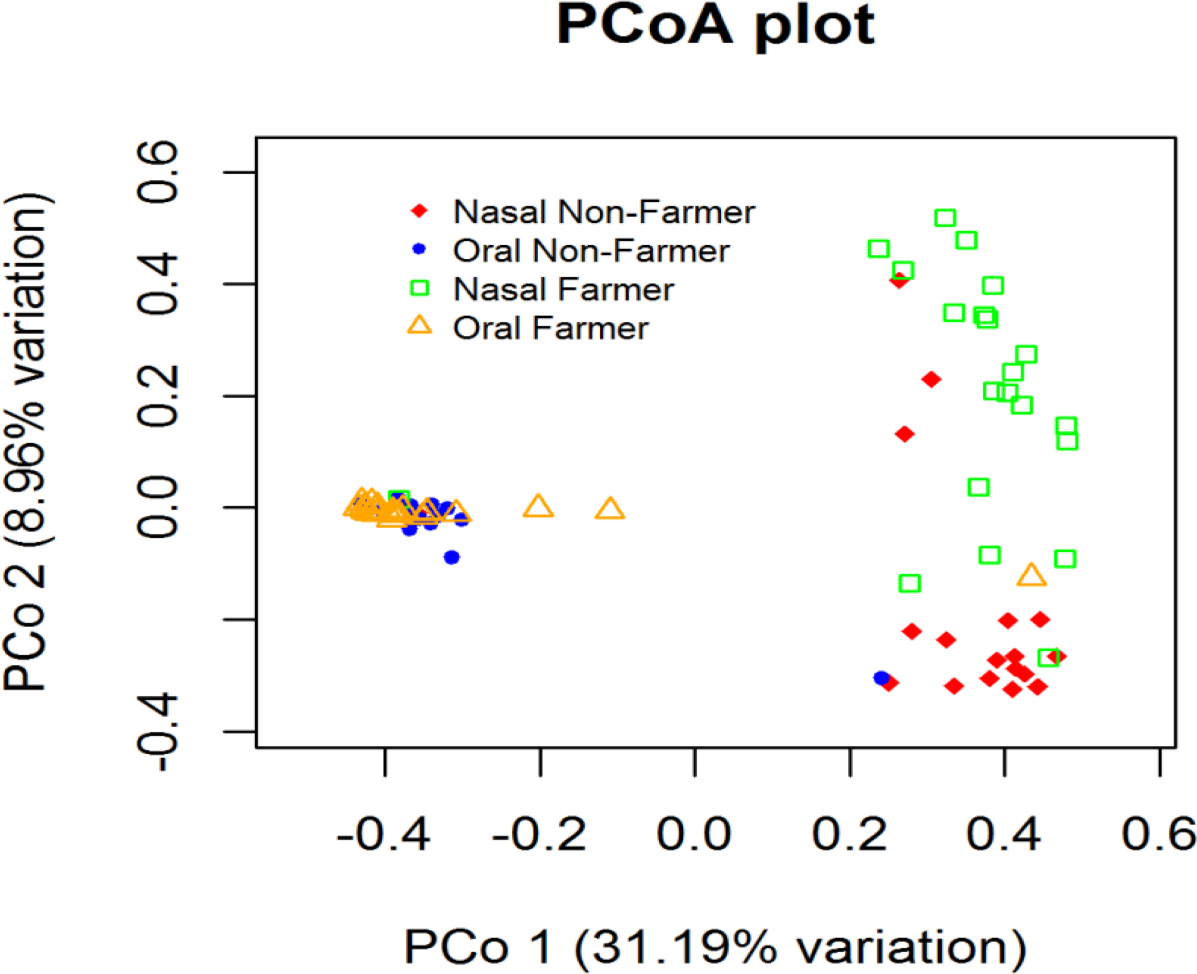
PCo a plot of microbiota in N_DF, N_NF, O_DF, and O_NF groups (N_DF = Nasal dairy farmer; N_NF = Nasal non-farmer; O_DF = Oral dairy farmer; O_NF = Oral non-farmer).

High beta diversity was seen between the oral and nasal microbiota regardless of the occupation status. There was no significant difference between oral microbiota of dairy farmers vs non-farmers. In PCoA, the first PCo represented 31.19% of the variance, and the second PCo represented 8.96% of the variance (Fig 2). Analysis of molecular variance (AMOVA) further showed the difference between N_DF and O_DF was statistically significant (AMOVA *p*-value <0.001) but not between O_DF and O_NF (AMOVA *p*-value 0.39) (Table 2).

**Table 2:** Analysis of molecular variance (AMOVA) between N_DF and N_NF (A) and AMOVA between O_DF and O_NF (B).

**A.**			
**N_DF versus N_NF**	**Among**	**Within**	**Total**
SS	1.59483	8.50051	10.0953
df	1	37	38
MS	1.59483	0.229743	
F statistics	6.9418		
*p*-value: <0.001*			
**B.**			
**O_DF versus O_NF**	**Among**	**Within**	**Total**
SS	0.179237	6.44629	6.62553
df	1	37	38
MS	0.179237	0.174224	
F statistics	1.02877		

*p*-value: 0.398

N_DF = Nasal dairy farmers; N-NF = Nasal non-farmers; O_DF = Oral dairy farmers; O_NF = Oral non-farmers. SS = sum of squares due to the source; df = degrees of freedom in the source; MS = sum of squares due to the source

### Dominance of phylum Bacteroidetes in nares of dairy farmers

The major phylas in all four groups were Firmicutes, Actinobacteria, Proteobacteria, Bacteroidetes, and Fusobacteria (≥1% cut off). The relative phyla level composition of the microbiota of the nasal samples was distinguishable from the microbiota of the oral samples but the latter group similar in both the groups.

Further the nasal samples of dairy farmers had distinct nasal microbiomes at the phylum level compared to the non-farming cohort. The N_DF had a significantly higher relative abundance of Bacteriodetes than N_NF (*p*-value <0.0001). The N_DF group also had an abundance of rare phyla group (phyla represented by <1%) compared to N_NF (*p*-value = 1.53E-05).

**Fig 3:**
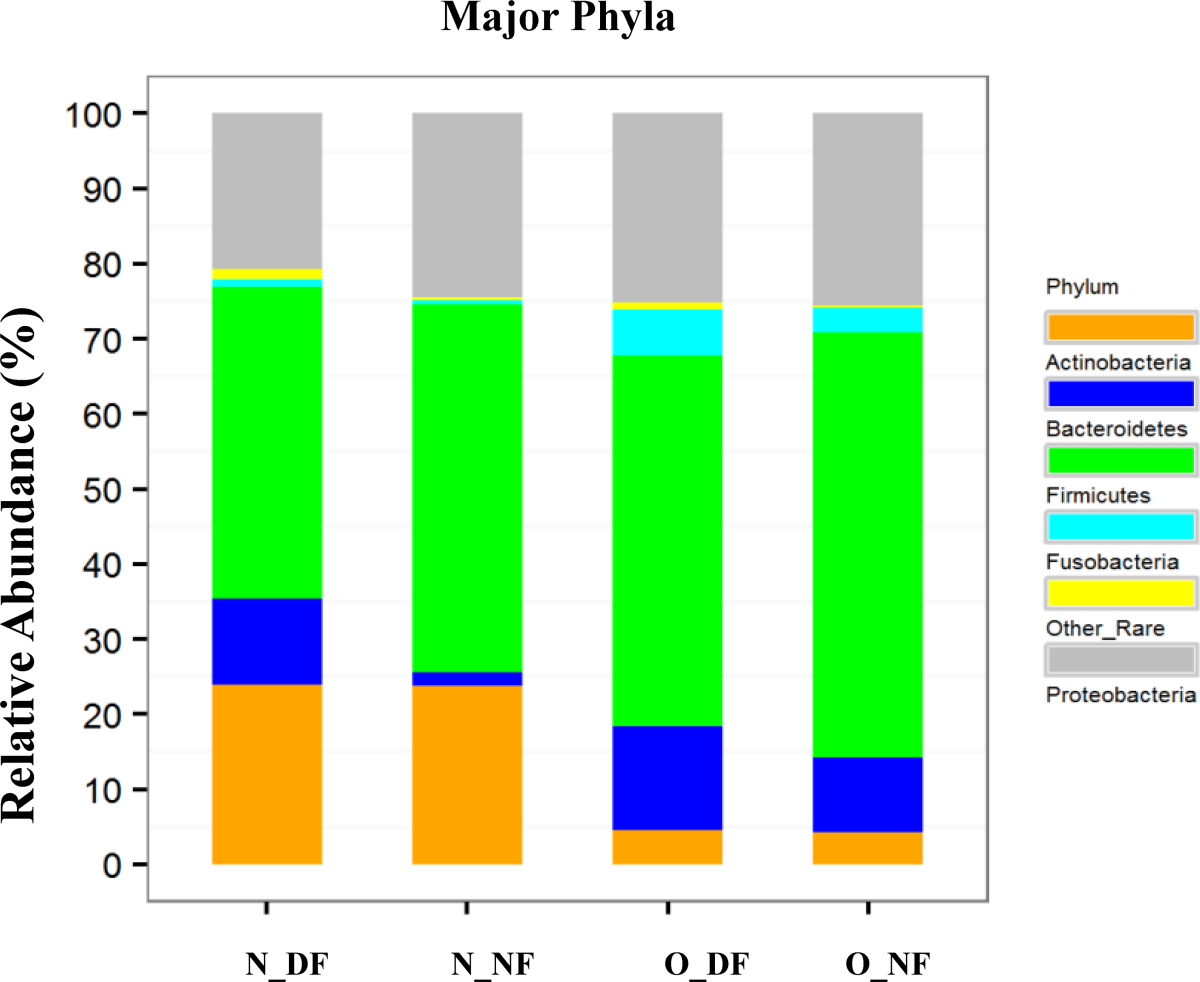
Relative abundance of major phyla in N_DF, N_NF, O_DF, and O_NF groups (N_DF = Nasal dairy farmer; N_NF = Nasal non-farmer; O_DF = Oral dairy farmer; O_NF = Oral non-farmer).

There was a significantly greater representation of Actinobacteria in nasal sample groups compared to the oral sample groups: N_DF vs. O_DF, *p*-value <0.0001; for N_NF vs. O_NF, *p*-value <0.0001. In contrast, both oral sample groups had a significantly greater representation of Fusobacteria than the nasal sample groups: N_NF vs. O_NF, *p*-value = 0.006 and N_F vs. O_F, *p*-value = 0.005 (Fig 3). Relative representation of Proteobacteria was similar in N_NF, O_DF, and O_NF; N_DF had a lower relative abundance of Proteobacteria compared to the other groups, although this was not statistically significant. Relative abundance of none of the phyla were significantly different when O_DF was compared with O_NF. All samples had a relatively large representation of Firmicutes (relative abundance range 43% [N_DF] to 57.3% [O_NF]).

### Carnobacteriaceae, Ruminococcaceae, and Lachnospiraceae dominated the nares of dairy farmers compared to non-farmers

**Fig 4:**
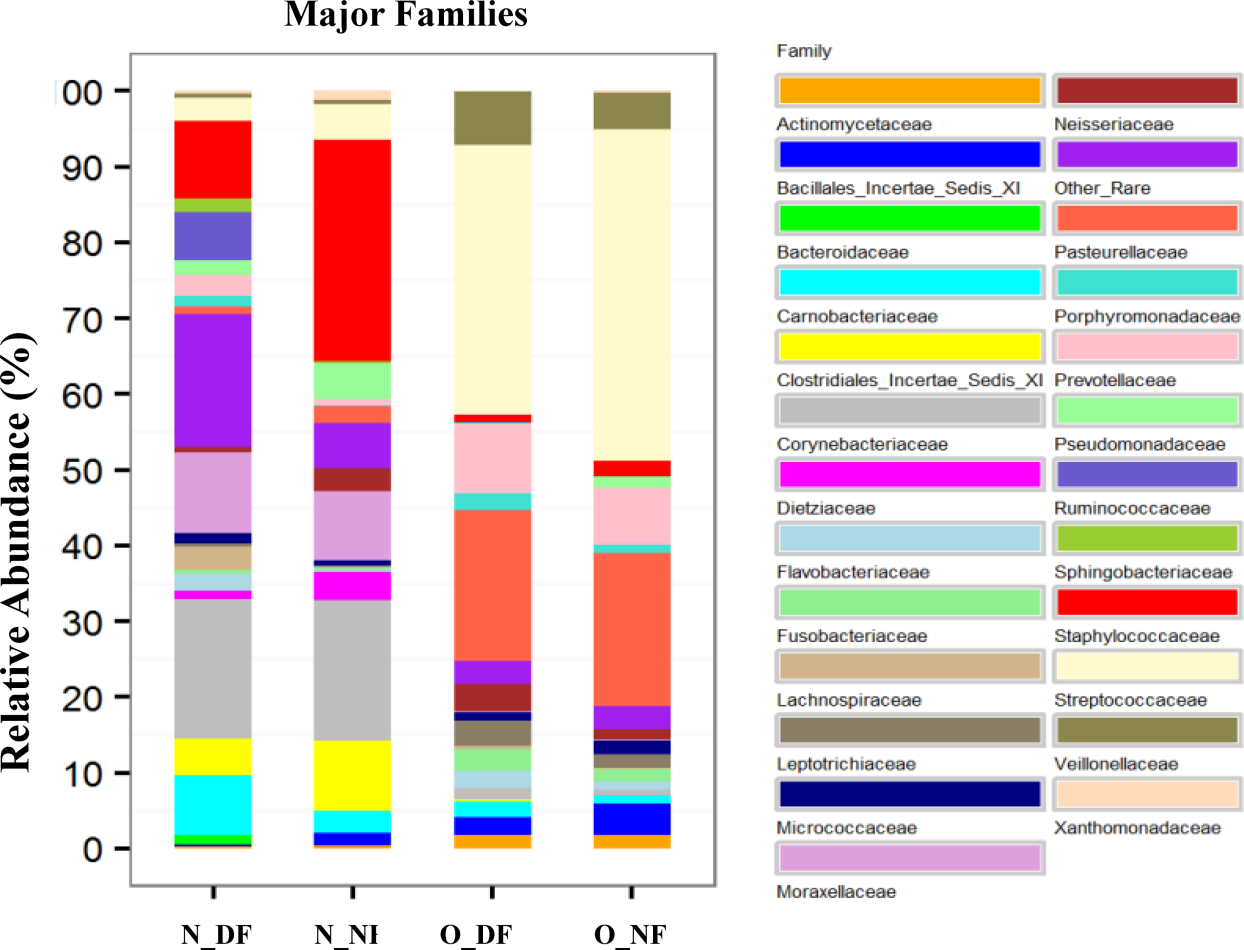
Relative abundance of major families in N_DF, N_NF, O_DF, and O_NF groups (N_DF = Nasal dairy farmer; N_NF = Nasal non-farmer; O_DF = Oral dairy farmer; O_NF = Oral non-farmer).

There were 24 major families identified from the four groups of samples (1% cut off). Families represented by <1% of the relative abundance were considered as rare-family groups (Fig 4). Eleven families significantly drove the differences between the microbiotas in the nares of dairy farmers vs non-farmers with Carnobacteriaceae, Ruminococcaceae, Lachnospiraceae, Flavobacteriaceae, Sphingobacteriaceae, Micrococcaceae, and Bacteroidaceae significantly higher in N_DF group while Staphylococcaceae, Pseudomonadaceae, and Dietziaceae were higher in N_NF group (Table 3). However, 21 families drove the difference between the nasal and oral microbiotas of dairy farmers at the family level. Of the 21 families, differences in abundance of 13 families (Corynebacteriaceae, Staphylococcaceae, Ruminococcaceae, Streptococcaceae, Streptococcaceae, Lachnospiraceae, Pseudomonadaceae, Sphingobacteriaceae, Bacteroidaceae, Pasteurellaceae, Dietziaceae, Veillonellaceae, and Xanthomonadaceae) in N_DF and O_DF were highly significant (*p*-value <0 .0001). Notably, Corynebacteriaceae, Moraxellaceae, Staphylococcaceae, Carnobacteriaceae, Ruminococcaceae, Clostridiales_Incertae_Sedis_XI, Lachnospiraceae, Pseudomonadaceae, Sphingobacteriaceae, Bacteroidaceae, and Dietziaceae were significantly higher in the N_DF group compared to Streptococcaceae, Prevotellaceae, Pasteurellaceae, Neisseriaceae, Veillonellaceae, Fusobacteriaceae, Leptotrichiaceae, Bacillales_Incertae_Sedis_XI, and Actinomycetaceae that were higher in the O_DF group (S2 Table). There was no significant difference between the O_DF and O_NF at the family level. Just like the differences between the N_DF and O_DF, significant differences were also noted between N_NF and O_NF (S3 Table). Streptococcaceae and Pasteurellaceae dominated the O_NF group, whereas Staphylococcaceae, Corynebacteriaceae, and Moraxellaceae dominated the N_NF group.

**Table 3:** Comparative relative abundance of microbiota of N_DF vs N_NF at the family level (1% cut off).

Family	N_DF	N_NF	*p*-value	Adjusted *p*-value	Sig
Corynebacteriaceae	19.20%	19.70%	9.18E-01	9.58E-01	
Moraxellaceae	10.80%	11.10%	9.62E-01	9.62E-01	
Staphylococcaceae	10.50%	29.80%	8.80E-03	2.64E-02	**
Carnobacteriaceae	8.0%	2.70%	2.66E-02	6.38E-02	*
Ruminococcaceae	6.20%	0.0%	1.32E-05	1.84E-04	***
Clostridiales_Incertae_Sedis_XI	4.50%	9.60%	5.68E-02	1.07E-01	
Streptococcaceae	3.50%	4.70%	6.26E-01	6.83E-01	
Lachnospiraceae	3.20%	0.10%	9.39E-05	4.51E-04	***
Prevotellaceae	2.90%	1.10%	6.64E-02	1.14E-01	
Flavobacteriaceae	2.40%	0.30%	2.66E-03	1.06E-02	**
Pseudomonadaceae	2.10%	5.10%	6.18E-03	2.12E-02	**
Sphingobacteriaceae	1.90%	0.20%	2.30E-05	1.84E-04	***
Micrococcaceae	1.40%	0.70%	9.89E-03	2.64E-02	**
Porphyromonadaceae	1.40%	0.10%	1.96E-05	1.84E-04	***
Bacteroidaceae	1.30%	0.0%	5.24E-05	3.15E-04	***
Pasteurellaceae	1.20%	2.0%	5.71E-01	6.83E-01	
Dietziaceae	1.0%	3.70%	4.64E-02	1.01E-01	*
Neisseriaceae	0.70%	3.10%	5.80E-02	1.07E-01	
Veillonellaceae	0.70%	0.50%	6.01E-01	6.83E-01	
Fusobacteriaceae	0.50%	0.30%	6.04E-01	6.83E-01	
Leptotrichiaceae	0.50%	0.20%	5.41E-01	6.83E-01	
Xanthomonadaceae	0.40%	1.40%	8.19E-02	1.31E-01	
Bacillales_Incertae_Sedis_XI	0.30%	1.30%	3.85E-01	5.44E-01	
Actinomycetaceae	0.20%	0.30%	3.30E-01	4.95E-01	

N_DF = Nasal Dairy Farmer; N_NF = Nasal non-Farmer, Sig = Significance

* p-value < 0.05

** p-value < 0.001

*** p-value < 1 E-4

### Hundreds of exclusive genera present in nares of dairy farmers

**Fig 5:**
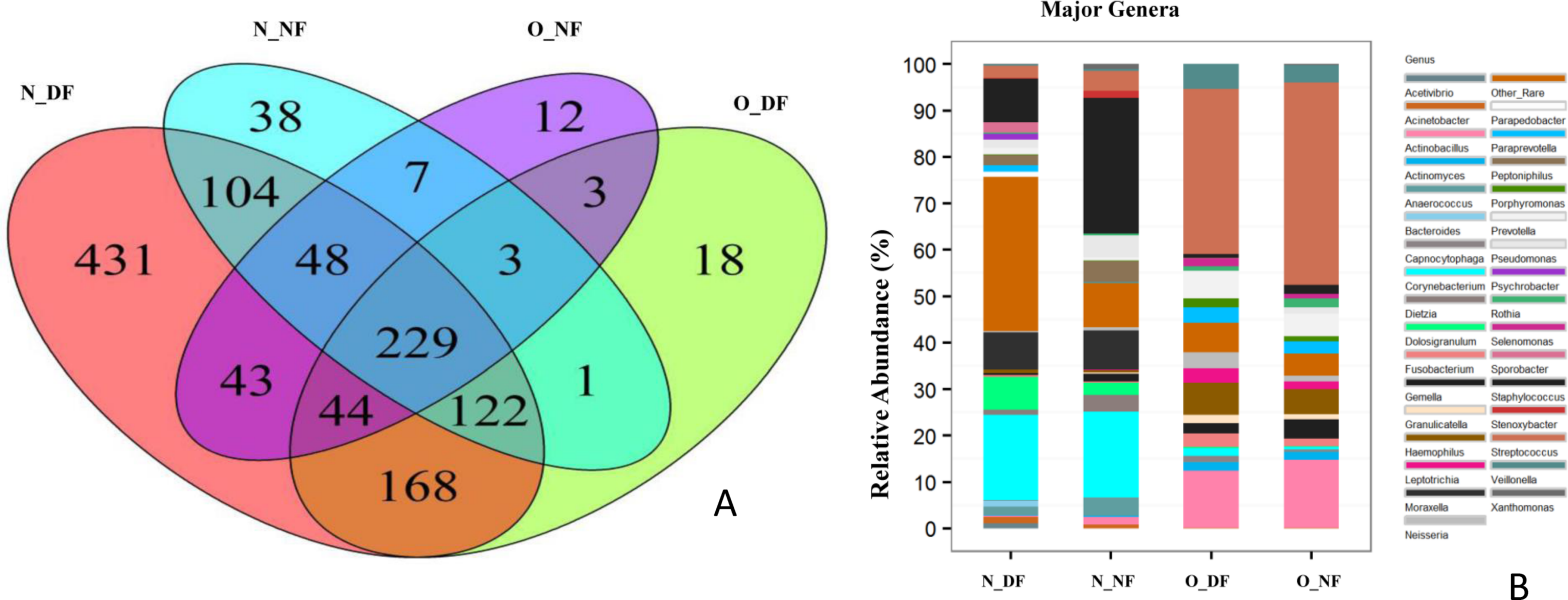
**A.** Venn diagram of number of common and exclusive phyla represented in N_DF, N_NF, O_DF, and O_NF groups (N_DF = Nasal dairy farmer; N_NF = Nasal non-farmer; O_DF = Oral dairy farmer; O_NF = Oral non-farmer). **B.** Relative abundance of major genera in N_DF, N_NF, O_DF, and O_NF groups (N_DF = Nasal dairy farmer; N_NF = Nasal non-farmer; O_DF = Oral dairy farmer; O_NF = Oral non-farmer).

The nasal microbiota of the dairy farmers group had 2.15 fold more genera (1189 genera vs. 552 genera) when compared to the nasal samples of non-farmers (no cut off). Similarly, the oral samples from the dairy farmers group harbored 1.5 fold more genera (588 genera vs. 389 genera) than the non-farmers group. The nasal microbiome of dairy farmers and non-farmers had 503 common genera (28.9%), whereas the oral samples of two groups shared 279 (28.6%) genera. The nasal and oral samples of dairy farmers had 563 (31.7%) genera in common between the two groups. Only 229 genera were common in all four groups (Fig 5A). The nasal samples from dairy farmers had the largest number of exclusive genera (n = 431), followed by N_NF (n = 38), O_DF (n = 18), and O_NF (n = 12) (Fig 5A). Of these, 32 genera were identified as major genera from the combined nasal and oral samples of dairy farmers and non-farmers group (1% cut off; Fig 5B). The top five most relatively abundant genera in nares of dairy farmers were *Corynebacterium* (19.7%), *Staphylococcus* (9.4%), *Moraxella* (8.1%), *Dolosigranulum* (7%), and *Streptococcus* (3.3%) compared to *Staphylococcus* (34.6%), *Corynebacterium* (19.9%), *Moraxella* (8.5%), *Pseudomonas* (5.1%), and *Peptoniphilus* (5%) in nares of non-farmers group. Of the major genera, nine were significantly different in their relative abundance between the two groups (N_DF vs N_NF), and these were *Staphylococcus*, *Sporobacter*, *Pseudomonas*, *Paraprevotella*, *Bacteroides*, *Psychrobacter*, *Parapedobacter*, *Acetivibrio*, and *Xanthomonas* between the nasal sample group of dairy farmers and non-farmers (Table 4). Non-farmers had higher relative abundance of *Pseudomonas*, *Xanthomonas*, and *Staphylococcus* than dairy farmers, while dairy farmers showed higher abundance of *Paraprevotella*, *Bacteroides*, *Parapedobacter*, and *Psychrobacter* than non-farmers. The relative abundance of *Staphylococcus* in N_NF was 34.7%, a difference of 3.6 fold compared to N_DF. However, the abundance of *Corynebacterium* was similar in the two groups. Furthermore, within the N_NF group, *Pseudomonas* and *Peptoniphilus* were among the top five most common genera, replacing *Dolosigranulum* and *Streptococcus* when compared to the N_DF in their relative abundance (Table 4). The top five relatively abundant genera in O_DF and O_NF were the same: *Streptococcus*, *Actinobacillus*, *Haemophilus*, *Prevotella*, and *Veillonella*.

**Table 4:** Differences in relative abundance of genera between N_DF and N_NF with the cutoff limit expanded to include genera (1% cut off).

Genus	N_DF (%)	N_NF (%)	*p*-value	Adjusted *p*-value	Sig
*Corynebacterium*	19.70%	20%	9.56E-01	9.56E-01	
*Staphylococcus*	9.50%	34.70%	3.59E-02	1.38E-01	*
*Moraxella*	8.20%	8.50%	9.53E-01	9.56E-01	
*Dolosigranulum*	7.10%	3%	1.21E-01	2.98E-01	
*Streptococcus*	3.40%	4.80%	6.16E-01	7.04E-01	
*Sporobacter*	2.20%	0.0%	3.74E-05	5.98E-04	***
*Peptoniphilus*	2.10%	5.0%	7.60E-02	2.21E-01	
*Pseudomonas*	1.90%	5.20%	3.55E-03	1.62E-02	**
*Anaerococcus*	1.80%	4.0%	1.13E-01	2.98E-01	
*Paraprevotella*	1.40%	0.10%	1.37E-03	7.31E-03	**
*Acinetobacter*	1.40%	0.80%	2.49E-01	4.42E-01	
*Prevotella*	1.30%	0.80%	2.82E-01	4.74E-01	
*Bacteroides*	1.30%	0.0%	1.24E-04	9.94E-04	***
*Psychrobacter*	1.20%	0.0%	4.59E-04	2.93E-03	***
*Parapedobacter*	1.20%	0.0%	7.78E-05	8.30E-04	***
*Acetivibrio*	1.10%	0.0%	1.31E-05	4.21E-04	***
*Dietzia*	1.10%	3.40%	5.04E-02	1.61E-01	
*Haemophilus*	0.50%	0.30%	6.71E-01	7.41E-01	
*Fusobacterium*	0.50%	0.40%	7.13E-01	7.61E-01	
*Gemella*	0.40%	1.90%	3.84E-01	5.37E-01	
*Veillonella*	0.30%	0.50%	4.65E-01	5.73E-01	
*Actinobacillus*	0.30%	2.10%	2.31E-01	4.34E-01	
*Rothia*	0.20%	0.50%	3.80E-01	5.37E-01	
*Neisseria*	0.20%	0.80%	4.02E-01	5.37E-01	
*Actinomyces*	0.20%	0.30%	3.87E-01	5.37E-01	
*Porphyromonas*	0.20%	0.10%	4.48E-01	5.73E-01	
*Stenoxybacter*	0.10%	1.50%	1.38E-01	3.15E-01	
*Leptotrichia*	0.10%	0.20%	5.76E-01	6.82E-01	
*Selenomonas*	0.10%	0.0%	3.67E-01	5.37E-01	
*Granulicatella*	0.10%	0.30%	2.14E-01	4.28E-01	
*Capnocytophaga*	0.0%	0.10%	1.55E-01	3.31E-01	
*Xanthomonas*	0.0%	1.20%	3.87E-02	1.38E-01	*

N_DF = Nasal dairy farmer; N_NF = Nasal non-Farmer, Sig = Significance

* *p*-value < 5.0E-02

** *p* -value < 1.0E-03

*** *p* -value < 1.0 E-04

The following genera were exclusively present in more than 50% of the N_DF group: *Acetanaerobacterium*, *Algoriphagus*, *Anaerofilum*, *Anaerostipes*, *Bergeyella*, *Erysipelothrix*, *Fibrobacter*, *Lutispora*, *5_genus_incertae_sedis*, *Ignavigranum*, *Coprobacillus*, *Clostridium_XVIII*, *Indibacter*, *Lachnospira*, *Oligella*, *Oleiphilus*, *Pseudaminobacter*, *Pseudoclavibacter*, *Paenalcaligenes*, *Gemmiger*, *Paraeggerthella*, *Rathayibacter*, and *Sharpea*. An exclusive genus is one that is present in only one group. However, both O_NF and O_DF had no exclusive genera present in more than one sample. Similarly, no exclusive genera were present in more than 10% of the subjects in the N_NF group.

At all taxonomic levels, the nasal microbiota of dairy farmers was the richest in bacterial diversity. The N_DF group was represented by five exclusive phyla: Nitorspirae, Aquificae, Caldiserica (formerly OP5), Fibrobacteres, and candidate phyla BRC1; 40 exclusive families, and 431 unique genera. The other three groups (N_NF, O_DF, and O_NF) did not harbor any unique phyla.

#### Lack of evidence of methicillin resistance gene, *mecA* in dairy farmer’s nasal microbiome

Only one of the 18 nasal (5.5%) samples from N_NF was positive for *mecA* gene. All other were negative for this genetic marker for methicillin resistance.

## Discussion

Although a large number of studies have identified and characterized commensal microbial diversity in humans in various disease states, no microbiome study has specifically analyzed the microbiota of dairy farmers to determine whether microbiome composition differs between farmers and non-farmers [38]. Here we showed that people living and working on dairy farms have a rich and distinct nasal microbiome compared to that of non-farmers. Richness and higher microbial diversity in dairy formers supports the biodiversity hypothesis, and living in urban environments could mean exposure to less diverse microbial flora. Lack of and exposure to biodiversity in people living in urban areas has been associated with increased incidences of allergic and inflammatory diseases [7]. Hanski et al. [7] noted lower environmental biodiversity in atopic individuals along with genetically less-rich gammaproteobacteria on their skin compared to healthy individuals. On the same note, the hygiene hypothesis explains that effective public health measures may have decreased exposure to microbiota that was once available. The decreased microbial diversity could be contributing to increases in chronic inflammatory conditions [5].

Dairy farmers have close, prolonged, daily interactions with their cows and the dairy environment, which includes dust and manure. The aerosols of such an environment contain high levels of bacteria and fungi and are different than the aerosols generated in non-dairy agricultural environments [39]. The skin, anterior nares, and to some extent the mouth, are important body sites that could easily acquire diverse microbes from their environment.

In our study, the nasal microbiome of both farmers and non-farmers were dominated by Firmicutes (43.1%; low G+C group), Actinobacteria (24.7%; high G+C group), and Proteobacteria (22%) at the phyla level. Non-farmers had significantly diminished Bacteriodetes, Tenericutes, and Verrucomicrobia, suggesting occupational exposures of farmers could be the cause of differences seen between the two groups.

A study, Frank et al. [40] reported a majority of nasal OTUs from healthy individuals belonged to Actinobacteria (69% of sequences), Firmicutes (27%), and to a lesser extent, Proteobacteria (4%). Another study of nasal microbiota of healthy individuals reported dominance of Actinobacteria and Firmicutes [41]. While Actinobacteria tend to be soil dwellers and help in decomposing organic matter, Firmicutes are known to constitute the largest proportion of gut microbiota.

This enhanced microbial diversity in dairy farmers is presumably due in part to increased exposure to livestock and livestock-associated items (manure, feed, etc.) as compared to non-farmers, since some of these bacterial families, genera, and species that appear to be associated with livestock were detected in samples from dairy farmers. For example, members of Ruminococcaceae, which were significantly higher (Table 3, 0.026%, *p*-value <0.0001) in the nose of farmers, live in the bovine gut and are active plant degraders [42,43]. Another study, which determined the nasopharyngeal microbiota of feedlot cattle at entry day 0 and 60 days after, reported it was dominated by Proteobacteria and Firmicutes. And at 60 days, the predominant genera were *Staphylococcus*, *Mycoplasma*, *Mannheimia*, and *Moraxella* [43]. Not surprisingly, both *Staphylococcus* and *Moraxella* were observed in the nasal samples groups, although dairy farmers had significantly lesser burden of *Staphylococcus* in their nares. Species of *Staphylococcus*, specifically *S*. *aureus* and *S*. *epidermidis*, are common inhabitants of the human anterior nares. Our data suggested a relatively lower abundance of staphylococci in N_DF group, which could be due to colonization resistance associated with microbial competition [44]. *S*. *aureus* is well known as an opportunistic pathogen and can produce a variety of diseases in humans and animals [45,46]. Thus, one way higher microbial biodiversity can assist a host is through the ability to resist colonization of opportunistic pathogens on certain anatomic body sites. In our study, abundance of *Corynebacterium* was similar in both dairy farmers and non-dairy farmers, suggesting a less significant role for this genus in competing for *Staphylococcus* as has been reported in some studies [40,47,48]. Yan et al. [31] have presented evidence of specific interaction between *Staphylococcus aureus* and *Corynebacterium* species showing that high relative abundance of *C*. *pseudodiphtheriticum* was associated with lack or lower abundance of *S*. *aureus*, whereas *C*. *accolens* seem to promote growth of *S*. *aureus* in vitro [31].

We did not characterize species level classification in our study, therefore it remains to be seen if other genera that are significantly higher in N_NF (e.g. *Sporobacter spp*., *Paraprevotella spp*., *Bacteroides spp*., *Psychrobacter spp*., *Parapedobacter spp*.) could have also played a role in competing with staphylococci. The higher relative abundance of *Dolosigranulum* in N_NF did not reach statistical significance in our study, but it could have an inverse relationship with *S*. *aureus*, as *Dolosigranulum pigrum* has been reported as a predictor for lack of *S*. *aureus* colonization in the nose [49,50]. In addition to the role for S. epidermidis and Dolosigranulum species, a role for Lachnospiraceae should be explored in S. aureus non-carrier phenotype as suggested by Espinosa-Gongora et al. [51].

We noted that nasal samples had a greater degree of microbial diversity than oral samples, regardless of occupation. This difference in composition could be due to easier access of aerosols to nasal cavities than the mouth, which is a closable organ, therefore limiting environmental exposure. Nasal samples thus provide more meaningful information with respect to differences in the external environment of dairy farmers versus non-farmers.

The nasal samples from dairy farmers had the highest number of exclusive genera present than any other sample group. Additional research will be necessary to determine the potential impact of these large numbers of exclusive genera in the nasal microbiome with respect to overall health of the farmers. Another notable finding from this study was the lack of evidence of MRSA in the nasal samples of dairy farmers, although they had 9% relative abundance of staphylococci. A larger sample size will show the true burden of methicillin-resistance staphylococci in nasal microbiome of dairy farmers.

Microbiome composition varies by race/ethnicity, age, diet, and disease status, and these demographic differences may alter an individual’s microbiome to a greater or lesser extent than occupational exposures [9,16,17,52]. As we collected samples from a population over 98% Caucasian/white, we do not anticipate a large variation in microbiome composition due to variation by race/ethnicity in our cohorts. Within-group variation may occur with respect to the number of years of farming exposure and companion animal ownership. Wlasiuk and Vercelli [38] note that early childhood exposure to a farming environment is significantly associated with decreased likelihood of developing allergies and asthma, and that this protective effect can last into adulthood; therefore, non-farming individuals with early exposure to farming environments might still benefit from farming exposure in terms of microbiome development and composition [38]. Companion animal ownership, particularly dogs, is also associated with decreased likelihood of developing allergies and asthma [52].

The unique environmental exposures of dairy farmers are reflected in the greater abundance and diversity of commensal microbial species detected in their oral and nasal cavities as compared to other life styles. Future studies will be performed to analyze the relationships among the nasal and oral microbiomes of dairy farmers and non-dairy farmers with respect to the number and type of adverse health outcomes. Assuming enhanced microbial diversity is an indicator of overall good health, dairy farmers should have better health outcomes than non-dairy farmers and other professions, depending upon individual genetics and life-style choices.

A diverse microbiota in farmers might help in maintenance of immune homeostasis, which is a balance between anti-inflammatory immune response (steady state) and pro-inflammatory (required during infections) responses. However a dysbiosis or loss of microbial diversity might perturb the microbial balance needed for inflammatory and anti-inflammatory immune response. One of the limitations of this study is the lack of cattle microbiome data from the respective farms.

## Supporting information

S1 Table PCR primers, locations on chromosomes, and expected amplicon sizes for *mec* genes (from Reference # 37). (DOCX)

S2 Table Comparison of relative abundance microbiota at family level between N_DF and O_DF. This is the S2 Table legend. N_DF = Nasal farmer, O_DF = Oral Farmer; Sig = Significance* *p*-value < 5.0E-02, ** *p* -value < 1.0E-03, *** *p* -value < 1.0 E-04.
(DOCX)

S3 Table Comparison of relative abundance of bacterial families between N_NF and O_NF. This is the S2 Table legend: N_NF = Nasal non-Farmer; O_NF = Oral non-Farmer, Sig = Significance, *p-value < 5.0E-02, ** *p* -value < 1.0E-03, *** *p* -value < 1.0 E-04.
(DOCX)
