# Supplementary material for: The nasal microbiota of dairy farmers is more complex than oral microbiota, reflects occupational exposure, and provides competition for staphylococci
Source: PLoS One. 2017 Aug 29;12(8):e0183898. doi: 10.1371/journal.pone.0183898 (PMC5574581; doi:10.1371/journal.pone.0183898)
Supplement: S1 Table — (DOCX) [file pone.0183898.s001.docx]

| **Supplemental Table 1.** PCR primers, locations on chromosomes, and expected amplicon sizes for mec genes (From Reference # 37) | | | | |
| --- | --- | --- | --- | --- |
| ***mec* complex primers** | **Oligonucleotide sequence**  **(5′-3′)** | **Nucleotide positions** | **Annealing temp**  **(°C)** | **Amplicon size**  **(bp)** |
| ***mec*AF1** | **AGATGATAACACCTTCTACAC** | **46278-47148** | **48** | **870** |
| ***mec*AR1** | **CTAATAGATGTGAAGTCGC** |  |  |  |
| ***mec*AF2** | **AAATTTCATCTTACAACTAATG** | **45547-46350** | **48** | **803** |
| ***mec*AR2** | **TGGATAATCACTTGGTATATC** |  |  |  |
| ***mec*AF3** | **TGAAGATATACCAAGTGATTATC** | **44899-45571** | **48** | **672** |
| ***mec*AR3** | **CTCGTTACAGTGTCACTTTC** |  |  |  |
