# Supplementary material for: The nasal microbiota of dairy farmers is more complex than oral microbiota, reflects occupational exposure, and provides competition for staphylococci
Source: PLoS One. 2017 Aug 29;12(8):e0183898. doi: 10.1371/journal.pone.0183898 (PMC5574581; doi:10.1371/journal.pone.0183898)
Supplement: S2 Table — This is the S2 Table legend. N_DF = Nasal farmer, O_DF = Oral Farmer; Sig = Significance* p-value < 5.0E-02, ** p -value < 1.0E-03, *** p -value < 1.0 E-04. (DOCX) [file pone.0183898.s002.docx]

| **SUPPLEMENTAL TABLE 2. Comparison of relative abundance microbiota at family level between N_DF and O_DF** | | | | | |
| --- | --- | --- | --- | --- | --- |
| **Family** | **N_DF** | **O_DF** | ***p*-value** | **Adjusted**  ***p*-value** | **Sig** |
| Corynebacteriaceae | 19.20% | 1.40% | 5.01E-05 | 1.83E-04 | *** |
| Moraxellaceae | 10.80% | 0.20% | 3.17E-03 | 5.43E-03 | ** |
| Staphylococcaceae | 10.50% | 1.0% | 4.99E-04 | 9.98E-04 | *** |
| Carnobacteriaceae | 8.0% | 2.10% | 7.37E-03 | 9.83E-03 | ** |
| Ruminococcaceae | 6.20% | 0.0% | 1.31E-05 | 6.27E-05 | *** |
| Clostridiales_Incertae_Sedis_XI | 4.50% | 0.30% | 1.29E-02 | 1.54E-02 | * |
| Streptococcaceae | 3.50% | 36.50% | 1.52E-06 | 3.64E-05 | *** |
| Lachnospiraceae | 3.20% | 0.40% | 2.90E-04 | 6.34E-04 | *** |
| Prevotellaceae | 2.90% | 9.50% | 1.19E-02 | 1.50E-02 | * |
| Flavobacteriaceae | 2.40% | 2.10% | 6.99E-01 | 6.99E-01 |  |
| Pseudomonadaceae | 2.10% | 0.10% | 1.17E-05 | 6.27E-05 | *** |
| Sphingobacteriaceae | 1.90% | 0.10% | 7.42E-06 | 5.93E-05 | *** |
| Micrococcaceae | 1.40% | 1.0% | 2.82E-01 | 2.95E-01 |  |
| Porphyromonadaceae | 1.40% | 2.40% | 1.63E-01 | 1.78E-01 |  |
| Bacteroidaceae | 1.30% | 0.0% | 5.33E-05 | 1.83E-04 | *** |
| Pasteurellaceae | 1.20% | 20.30% | 5.31E-06 | 5.93E-05 | *** |
| Dietziaceae | 1.0% | 0.0% | 2.49E-04 | 6.34E-04 | *** |
| Neisseriaceae | 0.70% | 3.70% | 2.49E-04 | 8.74E-03 | ** |
| Veillonellaceae | 0.70% | 7.50% | 2.86E-04 | 6.34E-04 | *** |
| Fusobacteriaceae | 0.50% | 3.0% | 5.58E-03 | 8.37E-03 | ** |
| Leptotrichiaceae | 0.50% | 3.70% | 3.87E-03 | 6.19E-03 | ** |
| Xanthomonadaceae | 0.40% | 0.0% | 2.64E-04 | 6.34E-04 | *** |
| Bacillales_Incertae_Sedis_XI | 0.30% | 2.30% | 1.60E-03 | 2.95E-03 | ** |
| Actinomycetaceae | 0.20% | 1.90% | 1.46E-02 | 1.67E-02 | * |
| N_DF = Nasal farmer, O_DF = Oral Farmer; Sig = Significance* *p*-value < 5.0E-02, ** *p* -value < 1.0E-03, *** *p* -value < 1.0 E-04 | | | | | |
