# Supplementary material for: The nasal microbiota of dairy farmers is more complex than oral microbiota, reflects occupational exposure, and provides competition for staphylococci
Source: PLoS One. 2017 Aug 29;12(8):e0183898. doi: 10.1371/journal.pone.0183898 (PMC5574581; doi:10.1371/journal.pone.0183898)
Supplement: S3 Table — This is the S2 Table legend: N_NF = Nasal non-Farmer; O_NF = Oral non-Farmer, Sig = Significance, *p-value < 5.0E-02, ** p -value < 1.0E-03, *** p -value < 1.0 E-04. (DOCX) [file pone.0183898.s003.docx]

| **SUPPLEMENTAL TABLE 3. Comparison of relative abundance of bacterial families between N_NF and O_NF** | | | | | |
| --- | --- | --- | --- | --- | --- |
| **Family** | **N_NF** | **O_NF** | ***p*-value** | **Adjusted**  ***p*-value** | **Sig** |
| Staphylococcaceae | 29.80% | 2.20% | 4.54E-04 | 2.18E-03 | *** |
| Corynebacteriaceae | 19.70% | 0.60% | 2.68E-05 | 3.22E-04 | *** |
| Moraxellaceae | 11.10% | 0.20% | 6.57E-02 | 9.86E-02 |  |
| Clostridiales_Incertae_Sedis_XI | 9.60% | 0.10% | 2.92E-04 | 2.18E-03 | *** |
| Pseudomonadaceae | 5.10% | 1.80% | 1.59E-02 | 3.56E-02 | * |
| Streptococcaceae | 4.70% | 44.30% | 3.45E-07 | 8.29E-06 | *** |
| Dietziaceae | 3.70% | 0% | 8.05E-03 | 2.15E-02 | ** |
| Neisseriaceae | 3.10% | 1.40% | 1.81E-01 | 2.07E-01 |  |
| Carnobacteriaceae | 2.70% | 1.20% | 2.83E-01 | 3.09E-01 |  |
| Pasteurellaceae | 2% | 20.10% | 3.76E-04 | 2.18E-03 | *** |
| Xanthomonadaceae | 1.40% | 0.30% | 8.83E-02 | 1.12E-01 |  |
| Bacillales_Incertae_Sedis_XI | 1.30% | 5.30% | 1.69E-01 | 2.03E-01 |  |
| Prevotellaceae | 1.10% | 7.60% | 1.63E-02 | 3.56E-02 | * |
| Micrococcaceae | 0.70% | 2% | 7.84E-02 | 1.05E-01 |  |
| Veillonellaceae | 0.50% | 5% | 2.09E-03 | 8.36E-03 | ** |
| Actinomycetaceae | 0.30% | 1.70% | 2.47E-02 | 4.94E-02 | * |
| Fusobacteriaceae | 0.30% | 1.70% | 2.87E-03 | 9.84E-03 | ** |
| Flavobacteriaceae | 0.30% | 1.10% | 7.69E-03 | 2.15E-02 | ** |
| Sphingobacteriaceae | 0.20% | 0% | 7.01E-02 | 9.89E-02 |  |
| Leptotrichiaceae | 0.20% | 1.90% | 6.30E-02 | 9.86E-02 |  |
| Lachnospiraceae | 0.10% | 0.20% | 4.35E-01 | 4.54E-01 |  |
| Porphyromonadaceae | 0.10% | 1.30% | 3.86E-02 | 7.12E-02 | * |
| Ruminococcaceae | 0% | 0% | 4.85E-02 | 8.31E-02 | * |
| Bacteroidaceae | 0% | 0% | 9.10E-01 | 9.10E-01 |  |
| N_NF= Nasal non-Farmer; O_NF=Oral non-Farmer, Sig=Significance, **p*-value < 5.0E-02, **** *p* -value < 1.0E-03,  *** *p* -value < 1.0 E-04 | | | | | |
